# Metabolomics provides new information on the changes occurring in thyroid tumours

**DOI:** 10.1186/1756-6614-6-S2-A3

**Published:** 2013-04-05

**Authors:** Waldemar Balcerzak, S Deja, P Młynarz, A Ząbek, M Orczyk-Pawiłowicz, M Głód, T Dawiskiba, D Pawełka

**Affiliations:** 1 First Department and Clinic of General, Gastroenterological and Endocrinological Surgery, Wrocław, Poland; 2 Faculty of Chemistry, Opole University, Opole, Poland; 3 Department of Bioorganic Chemistry, Wrocław University of Technology, Wrocław, Poland; 4 Department of Chemistry and Immunochemistry, Wrocław, Poland; 5 Department and Clinic of Vascular, General and Transplantation Surgery, Wrocław, Poland

## 

Metabolomics is a part of systems biology dealing with the determination of qualitative and quantitative profile of low molecular weight compounds (metabolites) present in body fluids and tissues of living organisms. Metabolic composition is strongly dependent on the state of homeostasis and any deregulation should affect it. For this reason, there is now increased interest in metabolomics as a potential tool to support cancer research. At the same time the analysis of metabolic pathways involved in the process of carcinogenesis provides the possibility of a more complete understanding of the mechanisms that are critical for tumour biology.

In this study, 1H NMR measurements were performed for thyroid tumour tissue and healthy tissue homogenates and analyzed by chemometric manner. Multivariate analysis of the data using the PCA, PLS-DA and OPLS-DA methods allowed a precise separation from normal thyroid tissue of all tumours originating in both benign and malignant lesions. In addition, classification of nodular goiter, follicular adenoma and malignant tumours was possible with comparable efficacy.

